# Lysophosphatidylcholine Induces NLRP3 Inflammasome-Mediated Foam Cell Formation and Pyroptosis in Human Monocytes and Endothelial Cells

**DOI:** 10.3389/fimmu.2019.02927

**Published:** 2020-01-09

**Authors:** Rafael Corrêa, Luís Felipe Fonseca Silva, Dalila Juliana Silva Ribeiro, Raquel das Neves Almeida, Igor de Oliveira Santos, Luís Henrique Corrêa, Lívia Pimentel de Sant'Ana, Leonardo Santos Assunção, Patrícia T. Bozza, Kelly Grace Magalhães

**Affiliations:** ^1^Laboratory of Immunology and Inflammation, Department of Cell Biology, University of Brasília, Brasilia, Brazil; ^2^Laboratory of Immunopharmacology, Institute of Oswaldo Cruz—Fiocruz, Rio de Janeiro, Brazil

**Keywords:** lysophosphatidylcholine, foam cells, lipid droplet, inflammasome, NLRP3, atherosclerosis

## Abstract

Foam cells are specialized lipid-loaded macrophages derived from monocytes and are a key pathological feature of atherosclerotic lesions. Lysophosphatidylcholine (LPC) is a major lipid component of the plasma membrane with a broad spectrum of proinflammatory activities and plays a key role in atherosclerosis. However, the role of LPC in lipid droplet (LD) biogenesis and the modulation of inflammasome activation is still poorly understood. In the present study, we investigated whether LPC can induce foam cell formation through an analysis of LD biogenesis and determined whether the cell signaling involved in this process is mediated by the inflammasome activation pathway in human endothelial cells and monocytes. Our results showed that LPC induced foam cell formation in both types of cells by increasing LD biogenesis via a NLRP3 inflammasome-dependent pathway. Furthermore, LPC induced pyroptosis in both cells and the activation of the inflammasome with IL-1β secretion, which was dependent on potassium efflux and lysosomal damage in human monocytes. The present study described the IL-1β secretion and foam cell formation triggered by LPC via an inflammasome-mediated pathway in human monocytes and endothelial cells. Our results will help improve our understanding of the relationships among LPC, LD biogenesis, and NLRP3 inflammasome activation in the pathogenesis of atherosclerosis.

## Introduction

Atherosclerosis is caused by a proinflammatory process (1) mediated by the deposition of excess lipids in the arterial intima (2), resulting in the development of lesions in the arterial walls. These atherosclerotic lesions are fibro-fatty plaques in the intima of arteries (3) characterized by abundant cells with multiple lipid droplets (LDs) in the cytoplasm. Owing to this foamy morphology, these enhanced LD-containing cells are called foam cells. Foam cells are fundamental components of the establishment and progression of atherosclerosis and leukocyte recruitment to the site of the disease (4). Foam cell formation within the subendothelial atherosclerotic microenvironment is induced by internalization of extracellular modified low-density lipoprotein (LDL), including highly inflammatory oxidized LDL (oxLDL) (5–7). The major bioactive lipid component of oxLDL is the phospholipid lysophosphatidylcholine (LPC) (4, 8, 9). This lipid is thought to be responsible for many of the inflammatory effects of oxLDL and is reported to increase atherosclerotic lesions (9–11).

LPC is a bioactive phospholipid generated by various biological processes, including phospholipase A2 (PLA2)-catalyzed cleavage of phosphatidylcholine (PC) (12) and oxidation of LDL. LPC is highly abundant in human blood, possessing a wide range of proinflammatory activities and an important role in cellular signaling. Most circulating LPC is associated with albumin and oxLDL molecules. Owing to this association, LPC has a major function in atherosclerosis as a critical factor in oxLDL's atherogenic activity and induces cellular cholesterol efflux (13).

As an important immunomodulator molecule, LPC can induce foam cell formation by promoting LD biogenesis (14), leukocyte activation and recruitment (15), cytokine secretion (14–19), and macrophage polarization (20). LDs are important cellular activation markers that function as key synthesis and storage sites for cytokine and lipid mediator generation (14). It was believed that the presence of LDs in cells was only related to lipid storage and transport. However, LDs are now known to be highly regulated organelles involved in many aspects of metabolism and cell activation, including the inflammatory response (21). The main contents of LDs are neutral lipids, triacylglycerol, cholesterol ester, phospholipids, and various proteins, reflecting the lipid uptake by the cells and its consumption. LD accumulation occurs in different pathologies, such as obesity, liver steatosis, myopathy, and atherosclerosis (22).

In addition to LDs, high levels of cholesterol crystals are found in the cytoplasm of enhanced LD-containing cells or foam cells (23–26). The formation of cholesterol crystals has been shown to activate the NLRP3 inflammasome (27), leading to IL-1β release (28, 29), clearly linking cholesterol metabolism with innate immune activation. Inflammasomes are intracellular multiprotein complexes present in activated cells, where they are responsible for mediating the activation of inflammatory caspases (30). This protein complex assembly leads to caspase-1 autoprocessing, which results in the maturation of the proinflammatory cytokines pro-IL-1β and pro-IL-18 into their active forms, IL-1β and IL-18. In addition to this process, a lytic form of cell death known as pyroptosis, characterized by membrane pore formation, can also be induced (31).

The first signal for inflammasome activation is initiated through pattern recognition receptor (PRR) signaling, which can recognize pathogen-associated molecular patterns (PAMPs), causing NF-κB translocation from the cytoplasm to the nucleus and promoting the transcription of pro-IL-1β and pro-IL-18. A second signal, which can be mediated by danger-associated molecular pattern (DAMP) recognition, is necessary to activate pro-caspase-1 into its active form, caspase-1. Then, caspase-1 can cleave pro-IL-1β and pro-IL-18 to their active forms, IL-1β and IL-18 (32). These second signals include reactive oxygen species (ROS) production (33), potassium efflux (34), and lysosomal damage caused by cathepsin B release to the cytoplasm (35).

Dysregulated inflammasome activation has been deeply associated with the establishment and treatment of atherosclerosis (36–43). LPC induces the secretion of proinflammatory cytokines, such as IL-1β (44, 45), TNF-α (46), and IL-6 (47), but it is still unclear whether LPC can modulate inflammasome activation and whether this process is involved in LPC-induced LD biogenesis. Here, we characterized the cellular and molecular mechanisms involved in the regulation of NLRP3 inflammasome activation and pyroptosis induced by LPC and investigated the role of this process in LD biogenesis-associated foam cell formation.

## Materials and Methods

### Culture of THP-1 Cells and Human Umbilical Vein Endothelial Cells

THP-1, a human monocyte cell lineage, was cultured in Roswell Park Memorial Institute (RPMI) 1640 (Sigma-Aldrich) supplemented with 10% fetal bovine serum (FBS) (Gibco) and 50 μM of 2-mercaptoethanol. Human umbilical vein endothelial cells (HUVECs), a human endothelial cell lineage, were cultured in DMEM/F12 (Sigma-Aldrich) supplemented with 10% FBS. The cells were maintained at 37°C under a 5% CO_2_ atmosphere.

### Lysophosphatidylcholine Treatment and Inhibitors

All experiments were conducted using the phospholipid 1-palmitoyl-2-hydroxy-*sn*-glycero-3-phosphocholine (LysoPC 16:0) from Avanti Polar Lipids. LPC stock was diluted in ethanol and kept at −20°C. Before all the assays, LPC was dried with nitrogen, diluted in culture medium, and sonicated for 10 min. For some experiments, lipopolysaccharide (LPS) (500 ng/ml; Sigma-Aldrich) and ATP (1 mM; Sigma-Aldrich) were used as positive controls. Depending on the assay, the cells were also treated with pharmacological inhibitors: *N*-acetyl-l-cysteine (NAC), 5 mM (Sigma-Aldrich); Ac-YVAD-cho, 20 μM (Enzo Life Sciences); glyburide, 150 μM (Sigma-Aldrich); CA074-ME, 50 μM (Sigma-Aldrich); statin, 25 μM (Sigma-Aldrich); GW9662, 1 μM (Sigma-Aldrich); methyl-beta-cyclodextrin, 10 μM (Sigma-Aldrich); and PAb-hTLR2, 10 μg/ml (InvivoGen).

### MTT Assay

MTT assays were performed in THP-1 cells and HUVECs treated with different concentrations of LPC. The cells were seeded in a 96-well plate and treated with 0.1, 1, 10, 20, 50, and 100 μg/ml of LPC. After 24 h, the medium was replaced with 10% MTT solution (5 mg/ml, Sigma-Aldrich, USA) diluted in culture medium. The cells were incubated for 4 h at 37°C with 5% solution, and then the MTT solution was removed and replaced with 100 μl of DMSO. The plate was further incubated for 5 min at room temperature, and the optical density (OD) of the wells was determined using a SpectraMax M3 plate reader (Molecular Devices, USA) at a test wavelength of 570 nm.

### Bodipy Staining and Flow Cytometry Analysis

THP-1 cells and HUVECs were stained with a fluorescent lipophilic probe, BODIPY 493/503 (Life Technologies), which binds with intracellular neutral lipids present in LDs. The probe was diluted in phosphate-buffered saline (PBS) at 1:5,000 for 30 min. At the end of the incubation, the cells were washed with PBS and centrifuged at 1,500 RPM for 5 min at 4°C; this process was repeated 3×. The supernatants were discarded, and the cells were resuspended in 500 μl of PBS. Data from 10,000 events were acquired on a FACSVerse system (BD Biosciences) and were analyzed with FlowJo software.

### FLICA Staining and Flow Cytometry Analysis

THP-1 cells and HUVECs were stimulated with 1 μg/ml of LPC or LPS (500 ng/ml) + ATP (1 mM) for 1, 3, and 24 h at 37°C. After this incubation time, the cells were stained with the fluorescent inhibitor probe FAM-YVAD-FMK, present in the FAM FLICA Caspase-1 assay kit (ImmunoChemistry Technologies, USA), to label active caspase-1 enzyme in living cells. The probe was added to the medium at 1:30 for 30 min. At the end of the incubation, we recovered the suspension of cells in a 2-ml Eppendorf tube (Eppendorf Safe-lock tube 2 ml, Sigma-Aldrich) and added 1.7 ml of 1× Apoptosis Washer buffer present in the assay kit. The tubes were centrifuged at 1,500 RPM for 2 min at room temperature. After centrifugation, the supernatant was carefully discarded, and then 1 ml of 1× Apoptosis Washer buffer was added. The tubes were centrifuged again under the same conditions. At the end, the supernatants were discarded, and the cells were resuspended with 300 μl of 1× Apoptosis Washer buffer. The cells were then analyzed by flow cytometry (BD FACSCalibur, BD Bioscience) using the FL1-H channel. The analysis and histogram graphics were performed using FlowJo software.

### Membrane Pore Formation Assay

The kinetics of membrane pore formation in the THP-1 cells and HUVECs were evaluated by analyzing propidium iodide (PI) uptake in the stained cells. The cells were seeded in a black, clear-bottom 96-well plate in a medium without phenol red and 1 mg/ml of PI. Treated cells were incubated at 37°C, and PI was excited at 538 nm. The fluorescence emission was read at 617 nm every 5 min using a SpectraMax M3 plate reader (Molecular Devices, USA).

### Microscopy

For confocal microscopy, THP-1 cells and HUVECs were grown on 12-mm circular glass slides. After incubation with LPC, the cells were washed and fixed with 4% paraformaldehyde in phosphate buffer (pH 7.4) (30 min, room temperature). For intracellular cell staining, the cells were permeabilized with 1% Triton X-100 plus 2% bovine serum albumin (BSA) in PBS. The cells were stained with anti-HMGB1 antibody (1:400) and Alexa Fluor 546 (1:2,000) secondary Ab for HMGB1 translocation analysis or with the BODIPY probe (1:300) for LD biogenesis analysis. Cellular nuclei were stained with 4′,6-diamidino-2-phenylindole (DAPI) (1:5,000). The images were taken using a Leica TCS SP5 confocal microscope (Leica Microsystems).

### Cytokine Measurement

Cytokines in the cell-free supernatants from the THP-1 cells and HUVECs were detected by enzyme-linked immunosorbent assays (ELISAs) according to the manufacturer's instructions (eBioscience and R&D Systems).

### Lactate Dehydrogenase Release

Cell death was determined by measuring lactate dehydrogenase (LDH) activity in the cell supernatants using a commercial cytotoxicity assay (CytoTox 96 nonradioactive cytotoxicity assay; Promega, Madison, WI, USA).

### Western Blot Analysis

HUVECs were treated with different concentrations of LPC (1 and 10 μg/ml) for 1, 3, and 24 h. The proteins were extracted using lysis buffer (50 mM of Tris-HCl, 150 mM of NaCl, 5 mM of EDTA, and 1% Triton X-100) and Cocktail Protease Inhibitor (04693159001, Roche). The gel contained 12% polyacrylamide, and the transfer occurred using a semidry system. The membrane was blocked for 1 h and incubated overnight at 4°C with the following primary antibodies: rabbit recombinant monoclonal anti-pro Caspase1 + p10 + p12 (ab179515, Abcam), HMGB1 mouse monoclonal antibody (66525-1-Ig, ptglab), anti-IL-1β (ab2105, Abcam), and anti-GSDMD rabbit monoclonal antibody ([EPR19829], ab210070, Abcam). The membrane was then incubated for 1 h with a rabbit or mouse secondary antibody (111-035-006, Jackson ImmunoResearch). The loading control was an anti-beta actin antibody (A3854, Aldrich). The bands were revealed using chemiluminescence substrate (Westar Supernova XLS3L and XLS3P) with an Image Quant LAS 4000 system (GE Healthcare Life Sciences). The bands were analyzed with ImageJ software (Version 1.8).

### Statistical Analysis

GraphPad Prism 6.0 (GraphPad Software) was used for the statistical analyses. Multiple group comparisons were conducted using one-way ANOVA, followed by Tukey's test or Student's *t-*test, as appropriate, and a *p* ≤ 0.05 was considered significant.

## Results

### Lysophosphatidylcholine-Induced Foam Cell Formation in Human Monocytes Is Dependent on HMG-CoA Reductase, PPARγ, and Lipid Rafts

**Figure 1:**
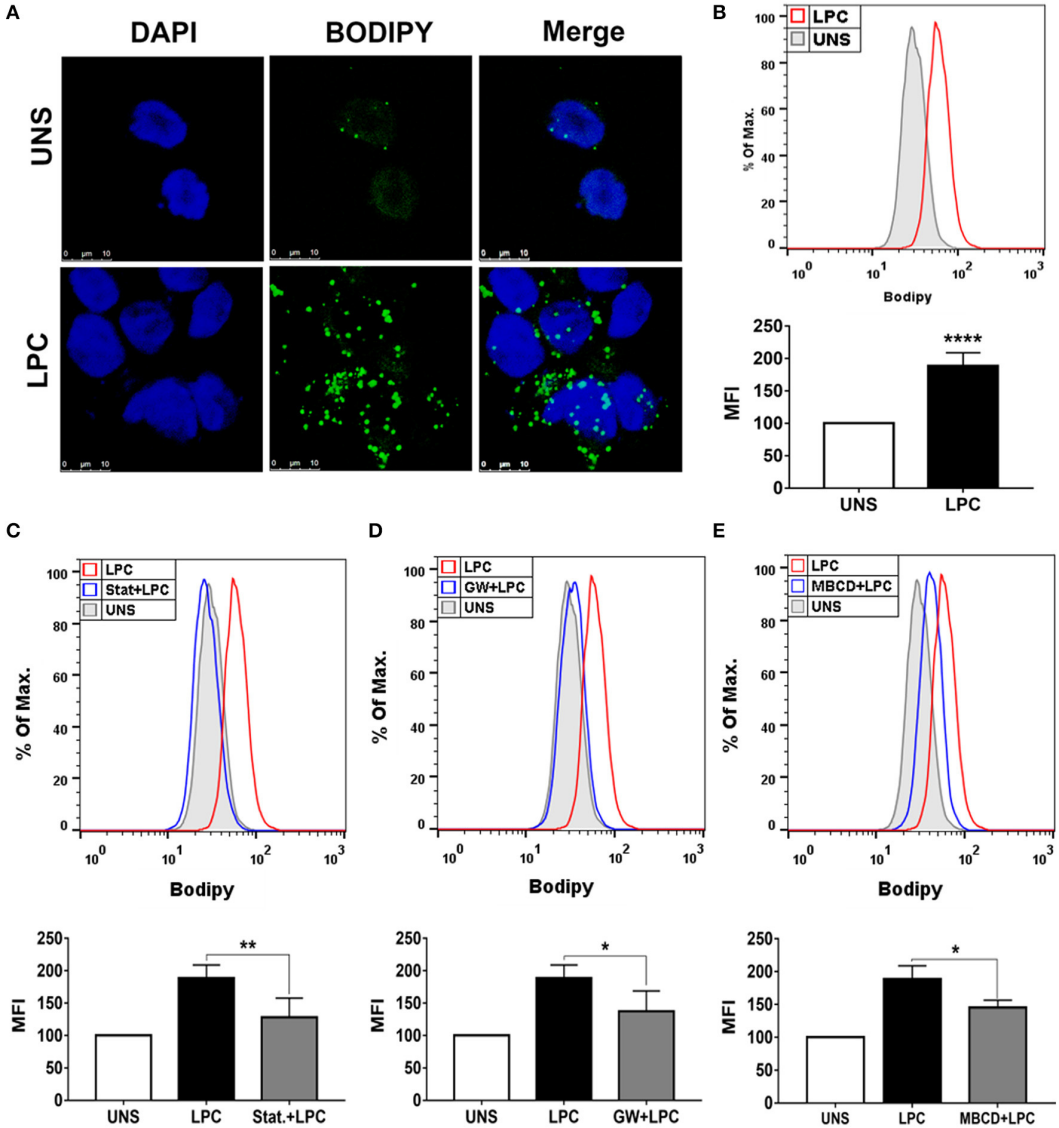
Lysophosphatidylcholine (LPC) induces foam cell formation in human monocytes through mechanisms dependent on HMG-CoA reductase, PPAR-γ, and lipid rafts. **(A)** Human monocytes were stimulated with 1 μg/ml of LPC, and after 24 h, lipid droplets were stained with the fluorescent probe BODIPY (green), and the nucleus was labeled with DAPI (blue). Images were taken by confocal microscopy. Scale bar, 25 μm. **(B)** Human monocytes were pretreated with **(C)** HMG-CoA reductase inhibitor (statin—Stat.), **(D)** antagonist of PPAR-γ [GW9662 (GW)], and **(E)** destabilizer of lipid rafts [methyl-β-cyclodextrin (MBCD)] for 1 h and stimulated with 1 μg/ml of LPC for 24 h. Lipid droplets were stained with BODIPY and analyzed by flow cytometry. Histograms are representatives of three independent experiments. Each bar graphic represents the mean fluorescence intensity (MFI), and bars show significant differences and represent the 95% confidence interval (*p < 0.05, **p < 0.01, and ****p < 0.0001) of the cells stimulated with LPC or UNS (unstimulated cells).

To verify whether LPC could induce foam cell formation in human monocytes, we treated these cells with 1 μg/ml of LPC for 24 h and analyzed LD biogenesis through confocal fluorescence microscopy and flow cytometry. LPC treatment increased LD formation in monocytes compared with those in untreated control cells, as shown by confocal microscopy images (Figure 1A). In addition, this result was quantitatively confirmed by flow cytometric analysis (see Supplementary Figure 1A), in which LPC induced increased LD biogenesis in human monocytes (Figure 1B). Furthermore, we investigated the mechanisms related to lipid metabolism involved in LPC-induced LD biogenesis. When HMG-CoA reductase, an important enzyme in cholesterol synthesis, was inhibited, a significant decrease in LPC-mediated LD production was observed (Figure 1C). Given that LPC induces PPARγ expression in macrophages (20), we investigated the role of PPARγ in LPC-induced LD biogenesis. Our results showed that inhibition of PPARγ decreases LD biogenesis in human monocytes stimulated with LPC (Figure 1D). Finally, we studied the role of lipid rafts in LD biogenesis induced by LPC. Disruption of lipid rafts induced a decrease in LD biogenesis in human monocytes stimulated with LPC (Figure 1E). The treatments did not reduce cell viability (see Supplementary Figure 2A).

### Lysophosphatidylcholine-Induced Foam Cell Formation in Human Endothelial Cells Is Dependent on HMG-CoA Reductase, PPARγ, and Lipids Rafts

**Figure 2:**
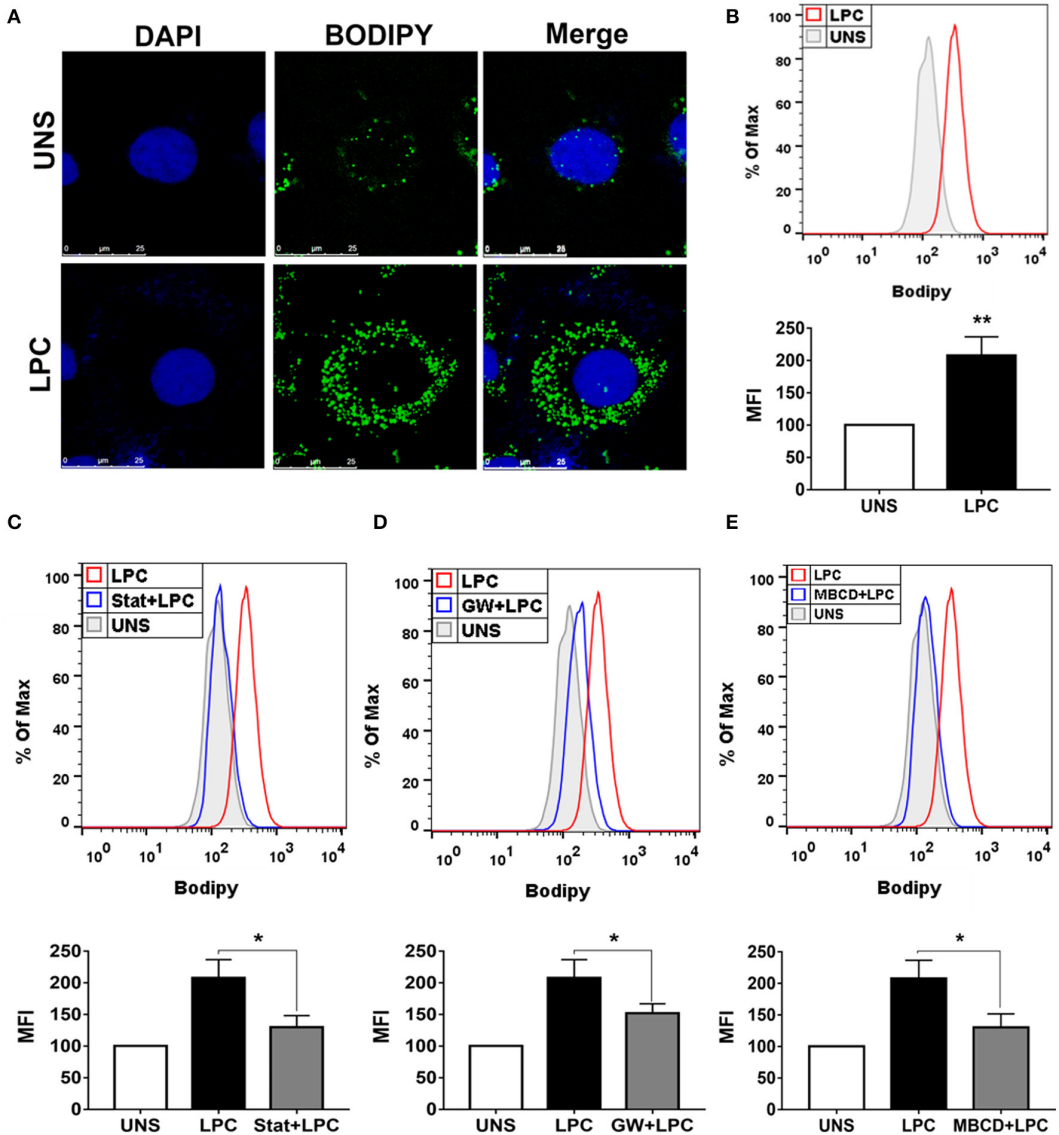
Lysophosphatidylcholine (LPC) induces foam cell formation in human endothelial cells through mechanisms dependent on HMG-CoA reductase, PPAR-γ, and lipid rafts. **(A)** Human endothelial cells were stimulated with 1 μg/ml of LPC, and after 24 h, lipid droplets were stained with the fluorescent probe BODIPY (green), and the nucleus was labeled with DAPI (blue). Images were taken by confocal microscopy. Scale bar, 25 μm. **(B)** Human endothelial cells were pretreated with **(C)** inhibitor of HMG-CoA reductase (statin—stat), **(D)** antagonist of PPAR-γ [GW9662 (GW)], and **(E)** destabilizer of lipids rafts [methyl-β-cyclodextrin (MBCD)] for 1 h and stimulated with 1 μg/ml of LPC for 24 h. Lipid droplets were stained with BODIPY and analyzed by flow cytometry. Histograms are representatives of three independent experiments. Each bar graphic represents the mean fluorescence intensity (MFI), and each bar graphic represents the mean fluorescence intensity (MFI), and bars show significant differences and represent the 95% confidence interval (*p < 0.05 and **p < 0.01) of the cells stimulated with LPC or UNS (unstimulated cells).

Endothelial cells play a critical role in vascular homeostasis and the development of atherosclerosis (48). Thus, the mechanisms involved in LPC-induced LD biogenesis were also investigated in human endothelial cells with the same experimental design mentioned above using human monocytes. LPC treatment increased LD formation in human endothelial cells compared with untreated control cells, as shown by confocal microscopy images (Figure 2A). In addition, this result was quantitatively confirmed by flow cytometric analysis (see Supplementary Figure 1B), in which LPC increased LD biogenesis in human endothelial cells (Figure 2B). Similarly, for human monocytes, we investigated the mechanisms related to lipid metabolism involved in the LPC-induced LD biogenesis in human endothelial cells. When HMG-CoA reductase (Figure 2C) and PPARγ (Figure 2D) were inhibited and lipid rafts were disrupted (Figure 2E), we observed a significant reduction in the LD biogenesis induced by LPC compared with that of the untreated cells stimulated with LPC that did not show decreased cell viability (see Supplementary Figure 2B).

### Lysophosphatidylcholine-Induced Caspase-1 Activation and Foam Cell Formation Is Dependent on Caspase-1 Activation

**Figure 3:**
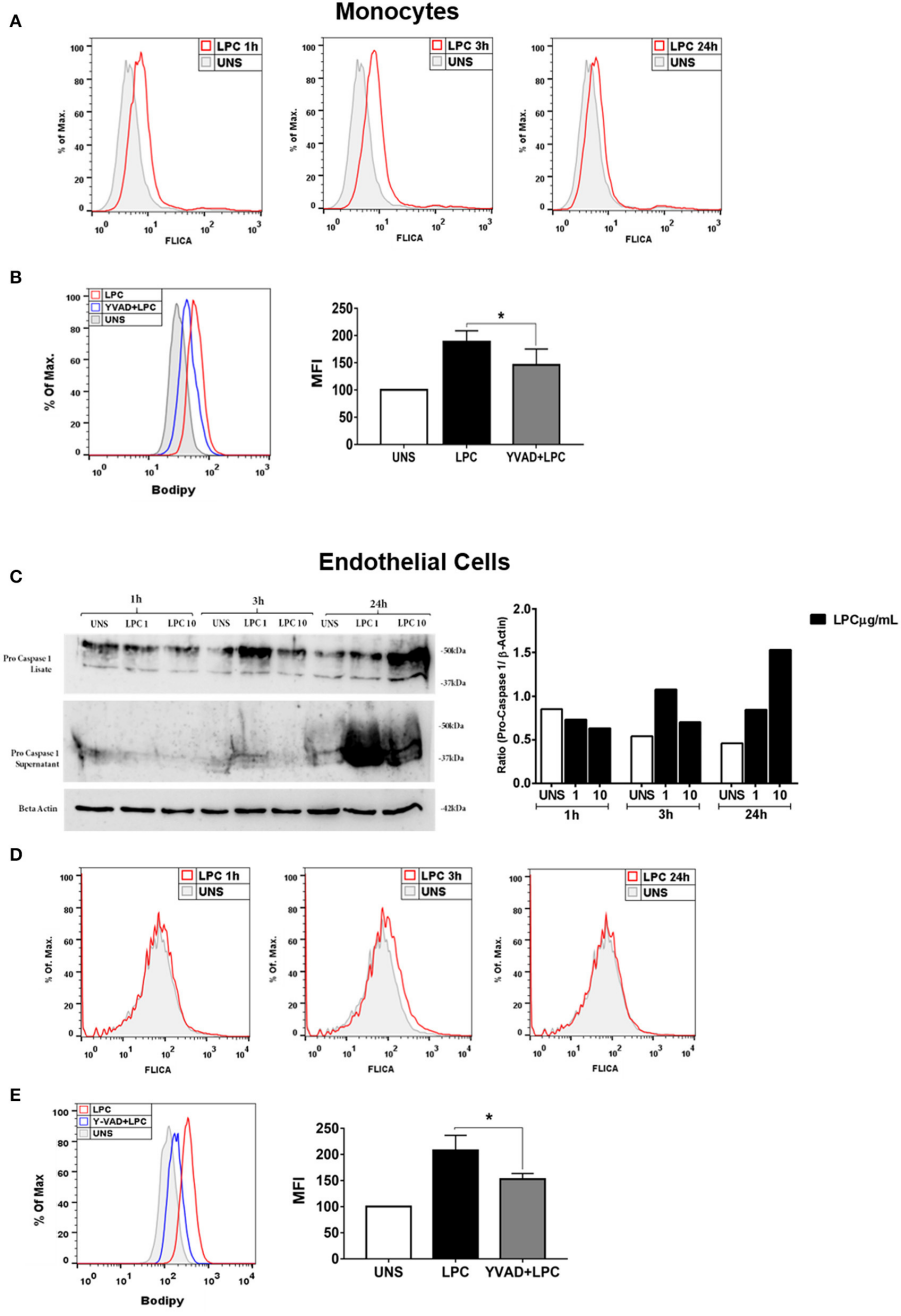
Lysophosphatidylcholine (LPC) induces activation of caspase-1 and foam cell formation in a caspase-1 activation-dependent manner. **(A)** Human monocytes and **(D)** human endothelial cells were stimulated with 1 μg/ml of LPC for 1, 3, and 24 h; the cells were stained with FAM-YVAD-FLICA to determine the caspase-1 activity and analyzed by flow cytometry. In addition, **(C)** the protein levels of pro-caspase-1 was examined by western blot analysis. Beta actin was used as an internal control. **(B)** Human monocytes and **(E)** human endothelial cells were pretreated with a caspase-1 inhibitor (Y-VAD) for 1 h and stimulated with 1 μg/ml of LPC for 24 h. Lipid droplets were stained with BODIPY and analyzed by flow cytometry. Histograms are representatives of three independent experiments. Each bar graphic represents the mean fluorescence intensity (MFI), and each bar graphic represents the mean fluorescence intensity (MFI), and bars show significant differences and represent the 95% confidence interval (*p < 0.05) of the cells stimulated with LPC or UNS (unstimulated cells).

The activity of the caspase-1 enzyme is critical for the processing and maturation of the IL-1β cytokine and the promotion of pyroptosis. Therefore, we investigated whether LPC could induce the activation of caspase-1. Human monocytes and human endothelial cells were stimulated with 1 μg/ml of LPC for different times. After the stimulation period, the cells were labeled with FLICA and analyzed by flow cytometry. The results demonstrated that LPC induced the activation of caspase-1 from 1 to 24 h in human monocytes (Figure 3A). However, in human endothelial cells, LPC induced activation of caspase-1 only at 3 h (Figure 3D). To confirm these results, we analyzed pro-caspase-1 via western blots, and increased expression was observed in cells stimulated with LPC after 24 h (Figure 3C). We also investigated the role of caspase-1 activation in foam cell formation. For this, both cell types were pretreated for 1 h with a caspase-1 inhibitor (Y-VAD) and stimulated with 1 μg of LPC for 24 h. After the stimulation period, the cells were labeled with BODIPY and analyzed by flow cytometry. We observed a significant decrease in LD biogenesis in the cells pretreated with the inhibitor of caspase-1 activity, compared with the untreated cells stimulated with LPC. Therefore, we showed that LPC-induced foam cell formation was dependent on caspase-1 activity in human monocytes (Figure 3B) and human endothelial cells (Figure 3E).

### Lysophosphatidylcholine-Induced IL-1β Release and Foam Cell Formation Are Dependent on Lysosomal Damage in Human Monocytes

**Figure 4:**
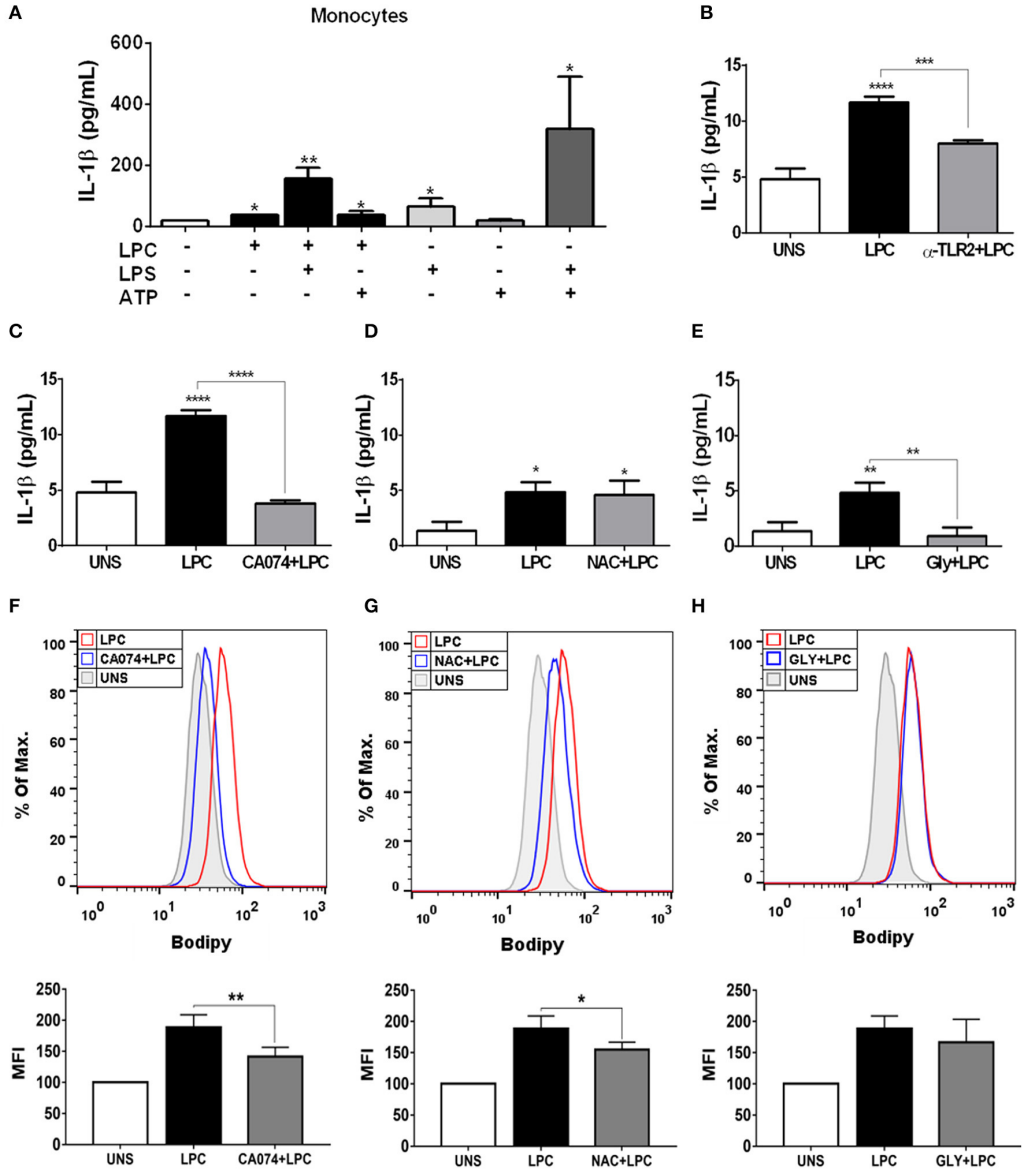
Lysophosphatidylcholine (LPC) induces IL-1β release and foam cell formation mediated by an inflammasome-dependent pathway in human monocytes. Human monocytes **(A)** were pretreated with LPS (500 ng/ml) 4 h before treatment with 1 μg of LPC for 24 h. After this treatment, the cells were treated with ATP (1 mM). One hour later, the supernatants were collected, and the levels of IL-1β were measured by ELISAs. Data are expressed as the average of triplicate wells. In addition, human monocytes were pretreated with different inhibitors: **(B)** TLR2 neutralizing antibody (α-TLR2), **(C)** cathepsin B inhibitor (CA-074), **(D)** reactive oxygen species (ROS) inhibitor [*N*-acetyl-l-cysteine (NAC)], and **(E)** inhibitor of potassium efflux [glibenclamide (GLY)] for 1 h and stimulated with 1 μg/ml of LPC for 24 h; the supernatants were collected, and the levels of IL-1β were measured by ELISAs. The bar graph represents one experiment performed twice in triplicate. Human monocytes were pretreated with different inhibitors **(F)** cathepsin B inhibitor (CA-074), **(G)** reactive oxygen species (ROS) inhibitor [*N*-acetyl-*L*-cysteine (NAC)], and **(H)** inhibitor of potassium efflux [glibenclamide (GLY)] for 1 h and stimulated with 1 μg/ml of LPC for 24 h, the lipid droplets were stained in cells with BODIPY and analyzed by flow cytometry. Histograms are representative of three independent experiments. Each bar graphic represents the mean fluorescence intensity (MFI), and bars show significant differences and represent the 95% confidence interval (*p < 0.05, **p < 0.01, ***p < 0.005, and ****p < 0.0001) of the cells stimulated with LPC or UNS (unstimulated cells).

Chronic inflammation of the arterial wall is a key element in the pathogenesis of atherosclerosis. Among the factors that trigger inflammation, the activation of the inflammasome and the consequent IL-1β and IL-18 secretion have been increasingly studied due to their atherogenic capacity (49). We investigated whether LPC could induce inflammasome activation through IL-1β release in human monocytes. To accomplish this, we treated and stimulated THP-1 cells as follows: (I) primed with 500 ng/ml LPS as the first signal of inflammasome activation and then stimulated with 1 μg/ml of LPC as the second signal of inflammasome activation; (II) stimulated with 1 μg/ml of LPC alone; and (III) stimulated with 1 μg/ml of LPC, the first signal, and treated with 1 mM of ATP as the second signal. Our results demonstrated that treatment with LPC alone induced a significant increase in IL-1β release in human monocytes. Moreover, this release was potentiated when the cells were primed with LPS. We also observed an increase in the IL-1β levels when LPC acted as a first signal and ATP acted as a second signal (Figure 4A).

Several mechanisms are associated with inflammasome activation and IL-1β secretion. To verify which mechanisms are involved in the secretion of IL-1β and foam cell formation by LPC, we pretreated human monocytes for 1 h with the following treatments: (A) TLR2 neutralizing antibody; (B) cathepsin B inhibitor; (C) Inhibitor of potassium efflux; and (D) ROS inhibitor. After treatment, the monocytes were stimulated with 1 μg/ml of LPC, and ~24 h after the treatments, the supernatants were collected, and the IL-1β release was measured by ELISAs. In parallel, the cells were labeled with BODIPY fluorescence probe, and LD biogenesis was quantitatively analyzed using flow cytometry. Our results showed that the induction of IL-1β secretion by LPC is dependent on TLR2 recognition, because there was a decrease in the IL-1β levels in the cells pretreated with the TLR2 neutralizing antibody, compared with the untreated cells stimulated with LPC (Figure 4B).

LPC-induced IL-1β secretion and foam cell formation were dependent on lysosomal damage because a significant reduction in IL-1β levels (Figure 4C) and a substantial decrease in LD biogenesis (Figure 4F) were observed in the cells pretreated with the cathepsin B inhibitor and stimulated with LPC, compared with the untreated cells stimulated with LPC (Figure 4C). Our results also showed that LPC induced LD biogenesis in an oxidative stress-dependent manner because BODIPY fluorescence staining decreased in the human monocytes treated with the ROS inhibitor (NAC) prior to LPC stimulation, compared with the untreated cells stimulated with LPC (Figure 4G). However, secretion of IL-1β induced by LPC was independent of the generation of ROS, because there was no decrease in IL-1β secretion in the cells pretreated with the ROS inhibitor, compared with the untreated cells stimulated with LPC (Figure 4D). When we inhibited the potassium efflux, there was a significant reduction in the IL-1β levels in the cells treated with the inhibitor, compared with the cells stimulated with LPC alone (Figure 4E), thus, IL-1β release induced by LPC is dependent on potassium efflux. However, there was no difference in the LD biogenesis in monocytes pretreated with the potassium efflux inhibitor prior to LPC stimulation and the cells stimulated with LPC alone, showing that foam cell formation was independent of potassium efflux in these cells (Figure 4H).

### Lysophosphatidylcholine-Induced Foam Cell Formation Is Dependent on the Second Signals of Inflammasome Activation in Human Endothelial Cells

**Figure 5:**
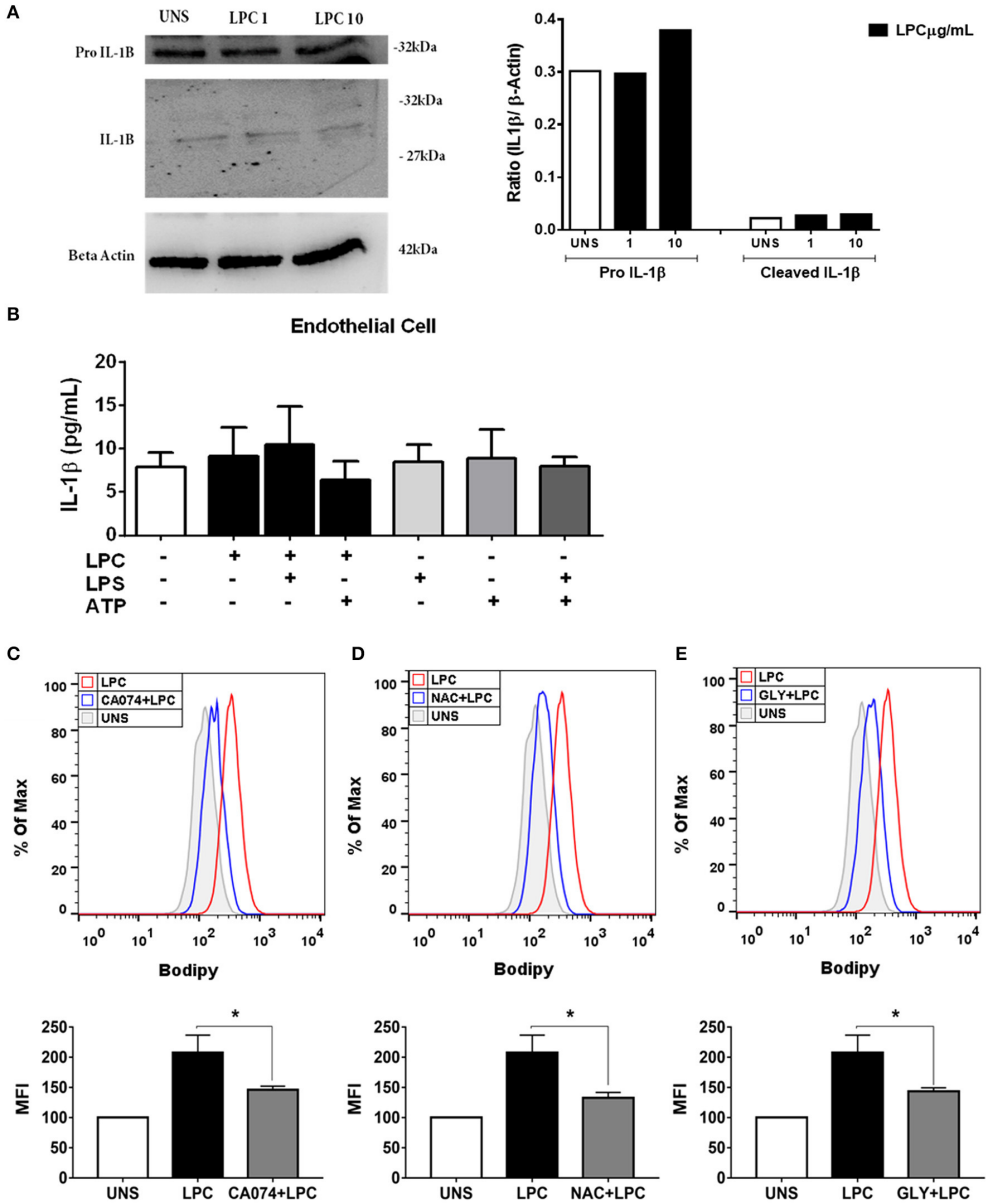
Lysophosphatidylcholine (LPC) induces foam cell formation mediated by an inflammasome-dependent pathway in human endothelial cells. Human endothelial cells were stimulated with 1 and 10 μg/ml of LPC. After 24 h, the protein levels of **(A)** pro–IL-1β and IL-1β in the supernatants or cell lysates were examined by western blot analysis. Beta actin was used as an internal control. Human endothelial cells **(B)** were pretreated with LPS (500 ng/ml) 4 h before treatment with 1 μg of LPC for 24 h. After this time, the cells were treated with ATP (1 mM). One hour later, the supernatants were collected, and the levels of IL-1β were measured by ELISAs. The bar graph represents one experiment performed twice in triplicate. In addition, human endothelial cells were pretreated with different inhibitors: **(C)** cathepsin B inhibitor (CA-074), **(D)** reactive oxygen species (ROS) inhibitor (*N*-acetyl-l-cysteine [NAC]), and **(E)** inhibitor of potassium efflux [glibenclamide (GLY)] for 1 h and stimulated with 1 μg of LPC for 24 h. Lipid droplets were stained with BODIPY and analyzed by flow cytometry. Histograms are representatives of three independent experiments. Each bar graphic represents the mean fluorescence intensity (MFI), and bars show significant differences and represent the 95% confidence interval (*p < 0.05) of the cells stimulated with LPC or UNS (unstimulated cells).

We investigated whether LPC could induce inflammasome activation through IL-1β release in human endothelial cells, and the expression of pro-IL-1β in the cells and IL-1β in the supernatant was evaluated by western blots. LPC did not induce alterations in the expression levels of pro-IL-1β and IL-1β (Figure 5A). To confirm the western blot data, we used the same experimental design mentioned above for monocytes for human endothelial cells. In contrast to the results in human monocytes, LPC did not induce IL-1β release in human endothelial cells (Figure 5B). In addition, our data demonstrated that LPC significantly induced the secretion of TNF-α compared to that of the unstimulated (UNS) control group of human endothelial cells (Supplementary Figure 3A) and human monocytes (Supplementary Figure 3C). The same proinflammatory effect was observed when LPC significantly induced IL-6 secretion compared with that in the UNS group of human endothelial cells (Supplementary Figure 3B) and human monocytes (Supplementary Figure 3D).

The mechanisms involved in LPC-induced LD biogenesis were also investigated in human endothelial cells with the same experimental design mentioned above. When we inhibited the release of cathepsin B, a substantial decrease in LPC-induced LD biogenesis was observed compared with that of LPC-only UNS cells (Figure 5C). Our results showed that LPC induced LD biogenesis in an oxidative stress-dependent manner. When we inhibited ROS production, a decrease in LPC-induced LD biogenesis was detected compared with that of LPC-only UNS cells (Figure 5D). However, unlike monocytes, when endothelial cells were treated with potassium efflux inhibitor, there was a significant decrease in LD biogenesis compared with that of cells stimulated with LPC alone, showing that the biogenesis of LD in endothelial cells is dependent on potassium efflux (Figure 5E).

### Pyroptosis Is Induced by Lysophosphatidylcholine in Human Monocytes and Endothelial Cells

**Figure 6:**
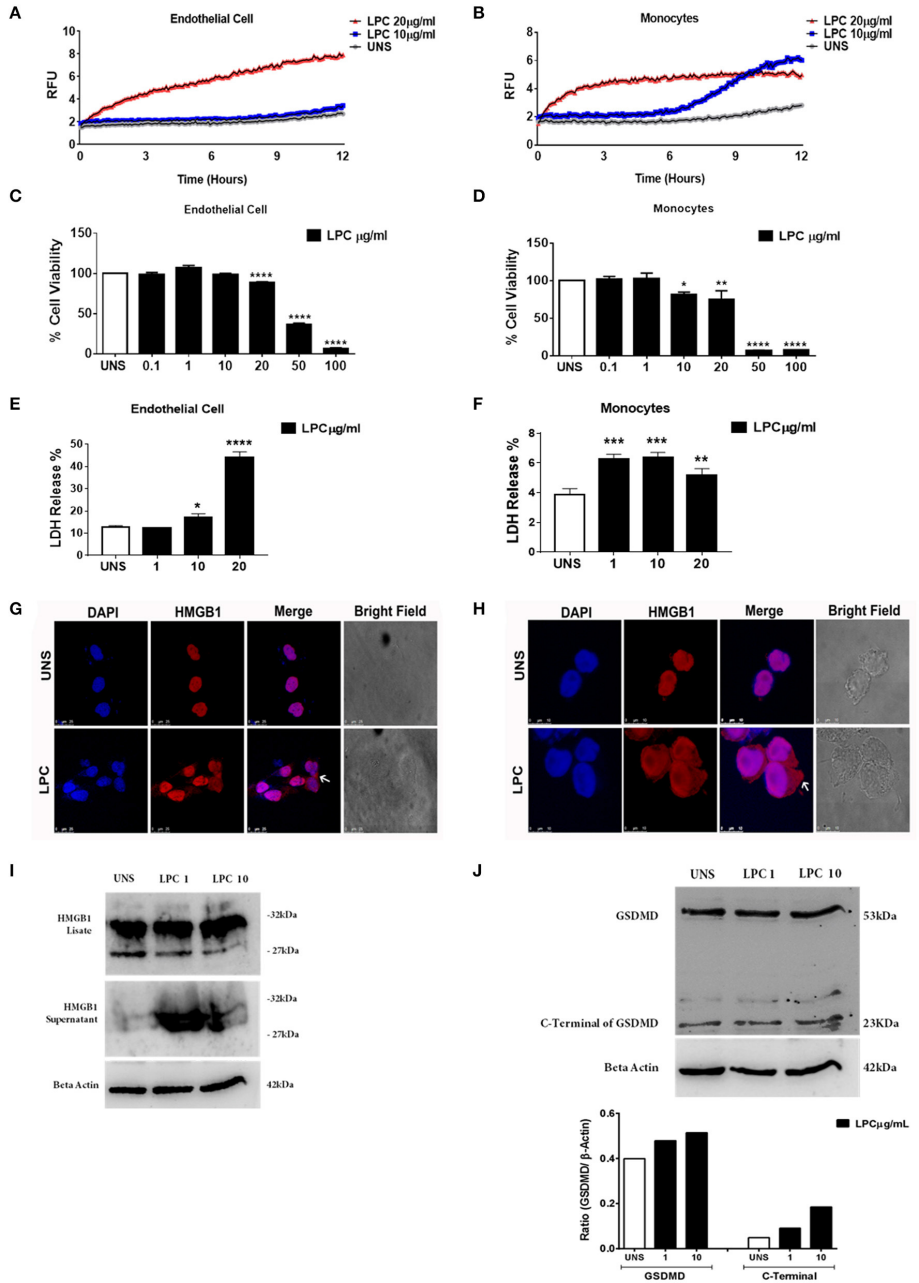
Lysophosphatidylcholine (LPC)-induced pyroptosis in human monocytes and human endothelial cells. **(A)** Human endothelial cells and **(B)** human monocytes were stimulated with 10 and 20 μg/ml of LPC. Pore formation was assessed fluorometrically in real time by the uptake of propidium iodide [relative fluorescence units (RFUs)]. The line represents one experiment performed twice in quintuplicate. **(C)** Human endothelial cells and **(D)** human monocytes were stimulated with different concentrations of LPC. Twenty-four hours later, the cells were incubated with MTT to evaluate the cytotoxicity. **(E)** Human endothelial cells and **(F)** human monocytes were stimulated with 1, 10, and 20 μg/ml of LPC. After 24 h, the LDH release was quantified. The bar graphs represent one experiment performed three times in triplicate. Each bar shows a significant difference and represents a confidence interval of 95% (*p < 0.05, **p < 0.01, ***p < 0.005, and ****p < 0.0001) with the unstimulated control. **(G)** Human endothelial cells and **(H)** human monocytes were stimulated with 10 μg/ml of LPC. After 18 h, the cells were stained with anti-HMGB1 conjugated with Alexa 546 and labeled with DAPI to visualize the nuclei. Images were taken by confocal microscopy. UNS: unstimulated cells. Bright field images were used as a morphology control. Scale bar, 25 and 10 μm. Human endothelial cells were stimulated with 1 and 10 μg/ml of LPC. After 24 h, the protein levels of **(I)** HMGB1 and **(J)** cleavage of GSDMD were examined by western blot analyses. Beta actin was used as an internal control.

To verify whether LPC triggers an inflammatory form of cell death, pyroptosis, we evaluated membrane pore formation in human monocytes and endothelial cells after treatment with LPC at two different concentrations. We found that only cells treated with 20 μg/ml of LPC exhibited an increase in membrane pore formation in human endothelial cells (Figure 6A) in the early hours of stimulation. This result corroborated the cell viability analysis and LDH release, where LPC significantly decreased cell viability at a concentration of 20 μg/ml (Figure 6C) and increased LDH release at 10 μg/ml (Figure 6E).

In human monocytes, treatment with LPC induced membrane pore formation at different concentrations and stimulation times. When we used the lowest concentration of LPC (10 μg/ml), membrane pore formation occurred ~7 h after treatment. However, when we used the highest concentration of LPC (20 μg/ml), membrane pore formation occurred within the first few hours of treatment (Figure 6B). This result was consistent with the cell viability analysis, where LPC at a concentration of 10 μg/ml significantly decreased cell viability after 24 h in human monocytes (Figure 6D). In addition, LPC at a concentration of 1 μg/ml increased LDH release after 24 h in human monocytes (Figure 6F).

HMGB1 is a high mobility, non-histone protein with a structural role in the chromosome architecture that is generally found in the nucleus. During inflammation, cellular activation, and cell death, these proteins translocate into the cytoplasm and are a marker of pyroptosis. In addition, the translocation of HMGB1 to the cytoplasm occurs after the activation of caspase-1 by different inflammasomes, leading to its subsequent secretion. After being secreted by cells of the immune system, HMGB1 functions as a DAMP, triggering an inflammatory response in several cells, leading to death by pyroptosis (50, 51). We also investigated whether LPC treatment in human monocytes and endothelial cells affected the translocation of HMGB1 to the cytoplasm. Our results showed that LPC (10 μg/ml) induced the translocation of HMGB1 to the cytoplasm in both endothelial cells (Figure 6G) and monocytes (Figure 6H). In addition, HMGB1 release and cleaved gasdermin D were detected by western blots. LPC increased HMGB1 release (Figure 6I) and gasdermin D cleavage in a dose-dependent manner in human endothelial cells (Figure 6J). These data confirmed that LPC induces pyroptosis in these cells.

## Discussion

Endothelial cells have an important role in the progression of atherosclerosis because they secrete chemokines and other substances that attract monocytes to the site of atherogenic development, in addition to overexpressing adhesion molecules that facilitate diapedesis (52–54). Circulating monocytes and resident macrophages are also fundamental cells in the development of atherosclerosis. These cells can phagocytize oxLDL molecules and cholesterol, storing these contents in cytoplasmic LDs, which are key organelles for cellular lipid metabolism, triggering differentiation of these cells into foam cells (55, 56). In this context, our data showed for the first time that endothelial cells can differentiate into foam cells after treatment with LPC, increasing the amount of LDs in their cytoplasm. Our results also confirmed that LPC can trigger an increase in LD biogenesis in human monocytes, leading to foam cell formation.

During the formation of foam cells, there is a large efflux of cholesterol and oxLDL that are stored in LDs. HMG-CoA reductase is an enzyme located in hepatic tissue that produces mevalonate, a small molecule used in the synthesis of cholesterol and other mevalonate derivatives, and is the target of drugs of the statin class used to lower cholesterol levels (57). Given the importance of cholesterol during the establishment and development of atherosclerosis, we decided to investigate the role of this enzyme in the LD biogenesis induced by LPC. Our data also showed that when HMG-CoA reductase is inhibited, LPC-induced LD biogenesis in monocytes and endothelial cells is decreased, corroborating the importance of cholesterol synthesis in LPC-induced foam cell formation.

PPARs play a key role in the regulation of cell differentiation, development, and lipid metabolism (58). The expression of PPARγ in macrophages is closely related to atherosclerosis. Using a murine model, Tontonoz and colleagues showed that PPARγ was highly expressed in the nucleus of foam cells isolated from atherosclerotic lesions (59). Therefore, we investigated the role of PPARγ in LPC-induced LD biogenesis. Our results showed that inhibition of PPARγ decreased LD biogenesis in both monocytes and endothelial cells. Our data are consistent with those described by Hou et al. in which LPC promotes cholesterol efflux in fat cells in a mechanism dependent on the activation pathway of PPARγ and LXFα associated with apoE (60).

In foam cells, cholesterol crystals can trigger NLRP3 inflammasome activation, leading to IL-1β release. Recently, Freeman et al. showed that LPC-induced IL-1β secretion in BMDMs is dependent on NLRC4, NLRP3, caspase-1, and ASC, whereas the non-canonical inflammasome-associated protein caspase-11 is not involved. In addition, LPC could induce NLRC4 and NLRP3 inflammasome activation in microglia and astrocytes in a caspase-1 activation-dependent manner (61, 62). LPC can induce IL-1β release in human monocytes (45). However, the mechanisms involved in this process have not yet been fully elucidated. The present work showed that LPC can induce IL-1β secretion by acting either as a first or second signal during inflammasome activation in human monocytes. This result corroborates the findings that electronegative LDL induces IL-1β secretion as a first or second signal for NLRP3 activation (63). Despite this result, we did not observe LPC-induced IL-1β secretion in human endothelial cells.

We also investigated the role of caspase-1 in LD biogenesis. Both the monocyte and endothelial cell results showed that when caspase-1 is inhibited, a decrease in LD formation induced by LPC is observed. In addition, our results showed a relationship between NLRP3 inflammasome activation pathways and foam cell formation. When the NLRP3 inflammasome is activated, it can promote the release of IL-1β and the induction of foam cell formation (64). Here, we verified that LD biogenesis is dependent on caspase-1 activation.

TLR2 is involved in the recognition of LPC (14, 65). Here, we showed that this recognition is essential for LPC-induced IL-1β secretion in human monocytes. The role of LPC-induced oxidative stress, through ROS generation, is already known (66). Here, we found that foam cell formation in both cells is dependent on ROS generation. However, the same signaling pathway did not mediate IL-1β secretion in human monocytes. Liu and colleagues demonstrated that oxLDL induces IL-1β secretion and foam cell formation through CD36, ROS generation, and NLRP3 activation (49). Our data corroborated this previous role of ROS generation in foam cell development. In addition, we demonstrated for the first time the mechanisms involved in foam cell formation in endothelial cells. By evaluating the role of other signals present during inflammasome activation, we demonstrated that IL-1β release, after treatment with LPC in human monocytes, is dependent on lysosomal damage and potassium efflux. These findings support the data from Stock et al., which demonstrated that blockage of potassium channels reduced the levels of IL-1β after treatment with LPC in microglia (44), and Liu et al. showed that blocking the release of cathepsin B decreased oxLDL-induced IL-1β secretion in monocytes (49). Analysis of the formation of foam cells showed that LPC-induced biogenesis is also dependent on potassium efflux and lysosomal damage in human endothelial cells. However, in human monocytes, this process was only dependent on lysosomal damage and was independent of potassium efflux. Consequently, we detected a direct relationship between NLRP3 inflammasome activation and IL-1β release in triggering LD biogenesis and foam cell formation.

Chang and colleagues demonstrated that LPC induces cytotoxicity/apoptosis and IL-8 production in human endothelial cells via mechanisms dependent on ROS, ATM/Chk2, ATR/Chk2, and PI3K/Akt signaling (67). Pyroptosis is a form of proinflammatory cell death and (68) can be mediated by inflammasome-dependent caspase-1 activation (69) or caspase-11 (70), which is responsible for the cleavage and/or maturation of gasdermin D (GSDMD) and membrane pore formation (71), IL-1β (72), IL-18 (73), HMGB1 (74), and LDH release (75). Pyroptosis has been shown to play important roles in cardiovascular diseases, such as atherosclerosis (76). In this work, we showed that LPC could induce pyroptosis in monocytes and endothelial cells owing to the increase in membrane pore formation and LDH released after treatment with high concentrations of LPC. These results agree with data shown by Lopez-Pastrana and colleagues, where LPC triggered pyroptosis of endothelial cells in a caspase-1-dependent manner in an ischemia model (77). However, we also identified the role of the HMGB1 protein in this LPC-induced pyroptosis of monocytes and endothelial cells.

**Figure 7:**
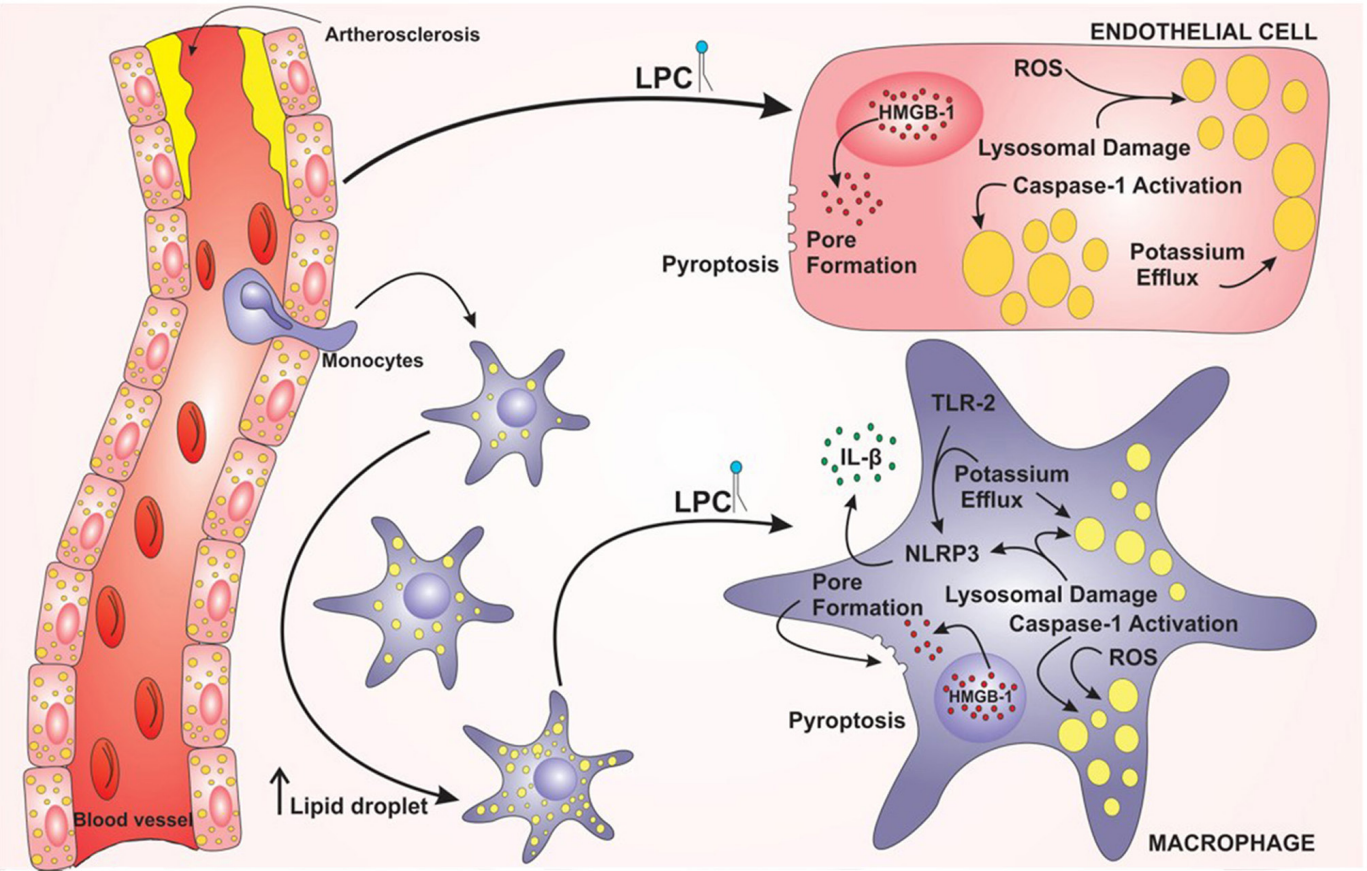
Model proposed for lysophosphatidylcholine (LPC)-mediated inflammasome activation and foam cell formation in human monocytes and endothelial cells. LPC plays a major role in the establishment and progression of atherosclerosis. LPC can increase the lipid droplets in macrophages and endothelial cells and promote inflammation through the activation of the NLRP3 Inflammasome. In addition, LPC can induce cell death by pyroptosis through the translocation of HMGB1 from the nucleus to the cytoplasm and pore formation.

Taken together, these results demonstrated that LPC can induce foam cell formation through LD biogenesis in human endothelial cells and human monocytes in an inflammasome-dependent pathway, as summarized in the graphical abstract (Figure 7). In addition, LPC induces the secretion of IL-1β in human monocytes but not in human endothelial cells. Surprisingly, LPC can induce pyroptotic cell death in human monocytes and human endothelial cells, and this process may be involved in the maintenance of the inflammation observed in the tissues adjacent to vascular regions and the pathology of atherosclerosis. Therefore, in the present work, we characterized different cellular and molecular mechanisms involved in foam cell formation and the creation of a microenvironment favorable for the establishment and maintenance of atherosclerotic plaques.

## Data Availability Statement

All datasets generated for this study are included in the article/Supplementary Material.

## Author Contributions

RC, LFS, DR, and KM conceived and designed the study. RC designed and performed the experiments, analyzed, and interpreted the data. RC, PB, and KM wrote and revised the manuscript. LFS, DR, RA, IS, LC, LPS, and LA participated in the data acquisition, analysis, and interpretation. All authors had critically revised and approved the final version of the manuscript.

### Conflict of Interest

The authors declare that the research was conducted in the absence of any commercial or financial relationships that could be construed as a potential conflict of interest.
