# Supplementary material for: Lysophosphatidylcholine Induces NLRP3 Inflammasome-Mediated Foam Cell Formation and Pyroptosis in Human Monocytes and Endothelial Cells
Source: Front Immunol. 2020 Jan 9;10:2927. doi: 10.3389/fimmu.2019.02927 (PMC6962110; doi:10.3389/fimmu.2019.02927)
Supplement: Supplementary file 1 [file Data_Sheet_1.pdf]

Supplementary Material

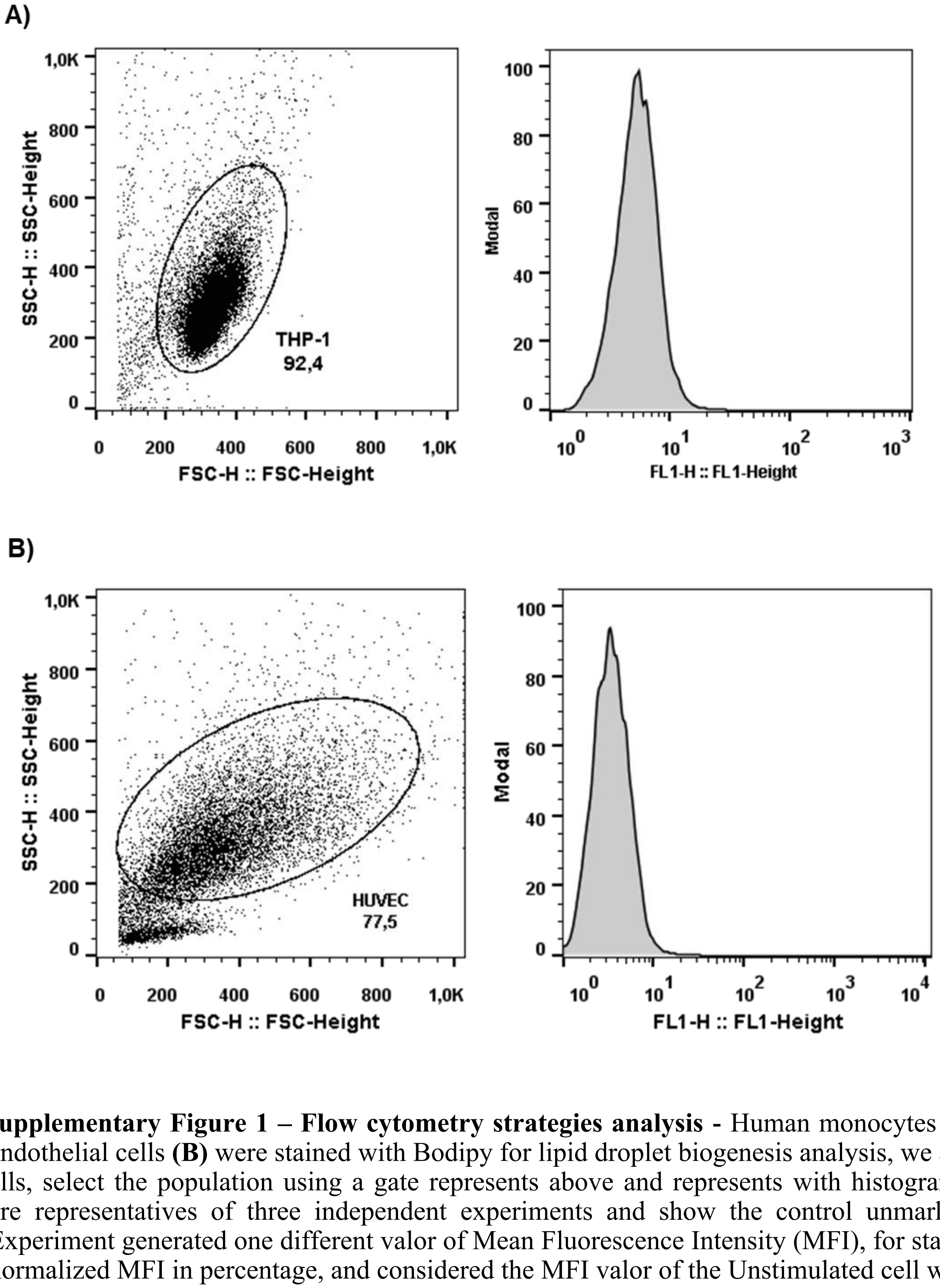

Supplementary Figure 1 – Flow cytometry strategies analysis - Human monocytes **(A)** and human endothelial cells **(B)** were stained with Bodipy for lipid droplet biogenesis analysis, we acquire 10000 c ells, select the population using a gate represents above and represents with histograms. Histograms are representatives of three independent experiments and show the control unmarked cells. Each Experiment generated one different valor of Mean Fluorescence Intensity (MFI), for statist analyses we normalized MFI in percentage, and considered the MFI valor of the Unstimulated cell with 100%.

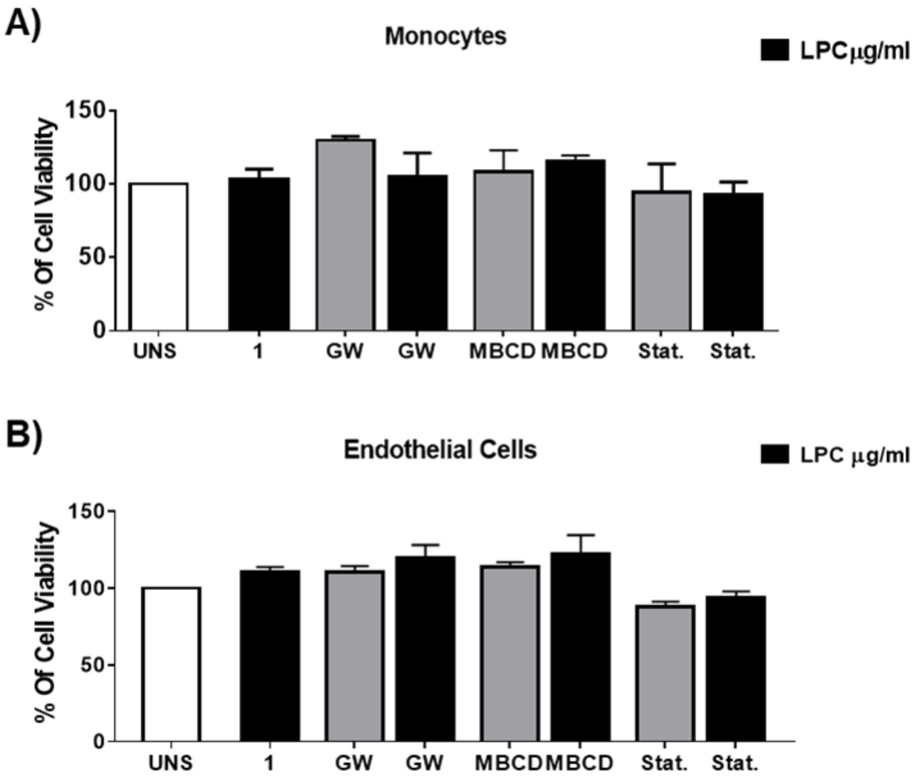

Supplementary Figure 2 – LPC and Inhibitors of HMG-CoA redutase, PPARγ and Lipid rafts treatments not decreased cell viability in human endothelial and human monocytes cells. Human monocytes **(A)** and human endothelial cells **(B)** were treated with Inhibitor of HMG-CoA redutase (Statin-Stat.); Antagonist of PPAR-γ (GW9662 – GW); Destabilizer of lipids rafts (Metil-β-ciclodextrina - MBCD); during one hour and stimulated with 1μg of LPC for 24h, cells were incubated with MTT in order to evaluate the cytotoxicity. Each bar represents statistical difference of bars representing confidence interval of 95% (* $p<0.05$, ** $p<0.01$, *** $p<0,005$ and **** $p<0.0001$) with the unstimulated control. Data are expressed as the average of triplicate wells.

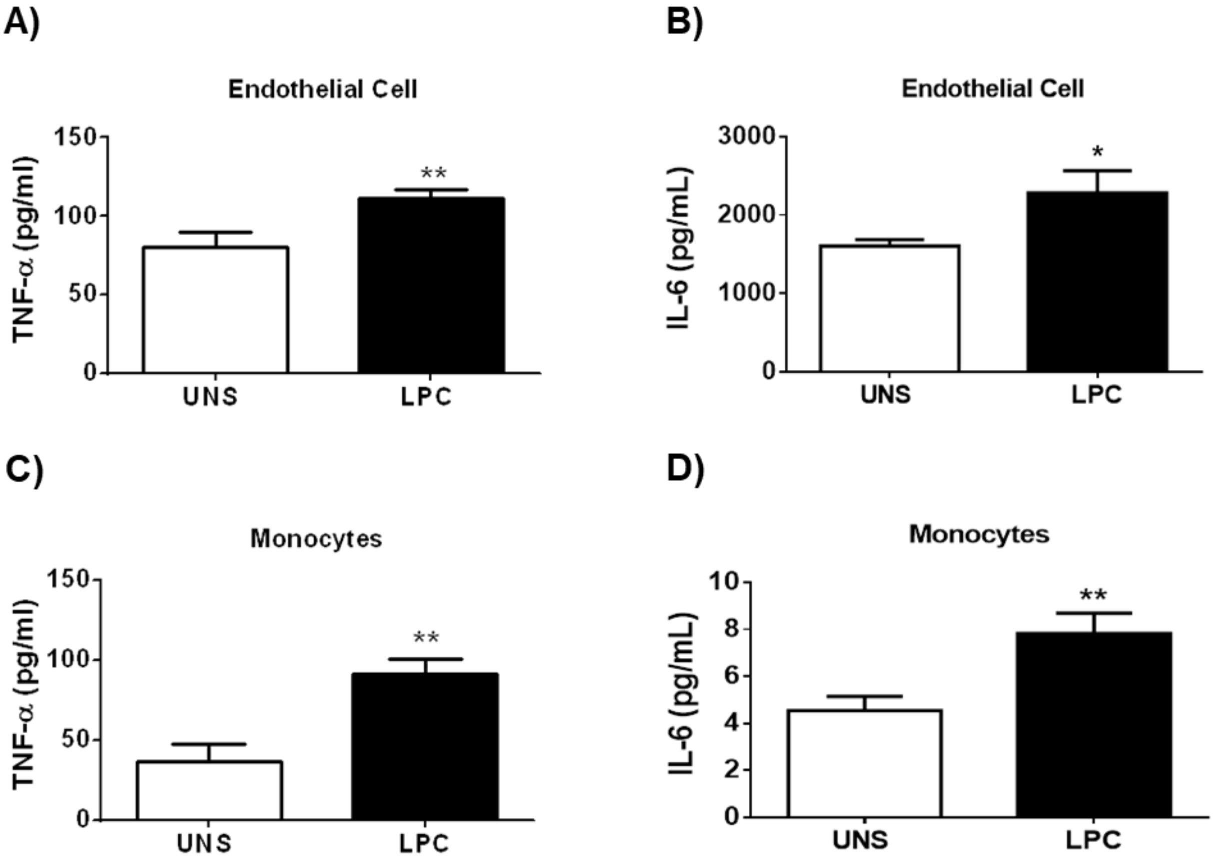

Supplementary Figure 3 - LPC induces IL-6 and TNF-α release in human monocytes and human endothelial cells. Human endothelial cells **(AB)** and human monocytes **(CD)** were treated with 1µg of LPC for 24h. After 24h supernatants were collected and the levels of IL-6 and TNF-α were measured by ELISA. UNS: Unstimulated cells. Data are expressed as the average of triplicate wells. The statistical difference of bars representing confidence interval of 95% (* $p<0.05$ and ** $p<0.01$) with the unstimulated control.
